# Development and Characterization of Bacterial Cellulose Reinforced with Natural Rubber

**DOI:** 10.3390/ma12142323

**Published:** 2019-07-21

**Authors:** Kornkamol Potivara, Muenduen Phisalaphong

**Affiliations:** Department of Chemical Engineering, Faculty of Engineering, Chulalongkorn University, Phayathai Road, Pathumwan, Bangkok 10330, Thailand

**Keywords:** bacterial cellulose, natural rubber, reinforcing, biodegradable polymers

## Abstract

Films of bacterial cellulose (BC) reinforced by natural rubber (NR) with remarkably high mechanical strength were developed by combining the prominent mechanical properties of multilayer BC nanofibrous structural networks and the high elastic hydrocarbon polymer of NR. BC pellicle was immersed in a diluted NR latex (NRL) suspension in the presence of ethanol aqueous solution. Effects of NRL concentrations (0.5%–10% dry rubber content, DRC) and immersion temperatures (30–70 °C) on the film characteristics were studied. It was revealed that the combination of nanocellulose fibrous networks and NR polymer provided a synergistic effect on the mechanical properties of NR–BC films. In comparison with BC films, the tensile strength and elongation at break of the NR–BC films were considerably improved ~4-fold. The NR–BC films also exhibited improved water resistance over that of BC films and possessed a high resistance to non-polar solvents such as toluene. NR–BC films were biodegradable and could be degraded completely within 5–6 weeks in soil.

## 1. Introduction

Pollution deriving from plastic materials is becoming one of the most prominent environmental concerns of recent years. Accumulation of plastic products in the environment has harmful effects on wildlife and their environment. Plastics dispersed in ocean ecosystems have become a major pollutant that has led to the direct deaths of marine animals. Therefore, developing renewable and biodegradable materials to replace conventional plastic materials is an increasingly important research area to reduce the level of plastic waste.

Thailand is the largest producer and exporter of natural rubber (NR) globally. Global natural rubber production in 2015 was 12.3 million tons, 92% of which was produced in the Asia-Pacific region. Thailand produced around 4.5 million tons and exported about 3.7 million tons in 2015 [1]. According to a recent release from the Association of Natural Rubber Producing Countries, global production of natural rubber was up in 2017 to ~13.3 million metric tons, but global consumption of NR dropped to ~12.9 million tons [2]. Therefore, research and development to expand commercial utilization of NR is required. NR latex (NRL) is a concentrated colloidal suspension produced by rubber trees. NRL is mainly composed of polyisoprene (poly(2-methyl-1,3-butadiene)). NR is a natural polymer of isoprene and is biodegradable. The most important property of NR is elasticity, which is the ability to return to its original shape and size. However, there are properties of NR that need to be improved, for example, hardness, Young’s modulus, and abrasion resistance [3]. Various natural fibers have been used as a reinforcement material in NR matrices such as sisal/oil palm hybrid fibers [4], pineapple fibers [5], coconut fibers [6], bamboo fibers [7], and grass fibers [8].

Bacterial cellulose (BC) is nanocellulose produced by bacteria, principally of genera Acetobacter, such as *Acetobacter xylinum*, in the form of interconnected networks of cellulose nanofibers. BC possesses unique properties such as high purity (excluding hemicellulose and lignin), high crystallinity, excellent mechanical strength (very high modulus and tensile strength), excellent biodegradability, high water uptake capacity (up to 100 cc/g), and excellent biological affinity [9]. The unique morphological alignment with Nano nonwoven structure of BC resulted in large surface area as compared with plant cellulose fibers or electrospun cellulose nanofibers [10]. With numerous advantageous characteristics, BC has found use across multiple industries. BC is adopted by the paper industry as an emulsion-stabilizing compound. Furthermore, BC can be applied in the medical area as artificial skin for patients with burns and ulcers [11], as artificial blood vessels [12], for drug delivery, and for tissue engineering and wound healing [13].

Although BC possesses numerous useful properties, one disadvantage is the low-breaking elongation. Conversely, NR is well-known for possessing excellent elastic properties. Reinforcements of NR achieved using graphene, carbon nanotubes, or nanocellulose fibers, such as bacterial cellulose, have been previously reported [14,15,16,17]. From our previous study, reinforcement of NR with BC was performed via a latex aqueous micro dispersion process and the films of BC–NR by incorporation of BC fibers into NR matrices demonstrated good water affinity and increased mechanical properties when compared with pure NR matrices [17]. However, the composite film of NR incorporated into BC matrices has not been reported so far. Herein, to obtain films with excellent mechanical properties of BC, characterized by high tensile strength, and NR, characterized by high elasticity, NR–BC films possessing a high degree of mechanical strength were developed by immersing BC pellicles into a diluted NRL suspension. Operational parameters such as temperature and NR concentration were studied to optimize the immersion process. NR–BC films were characterized for their chemical and mechanical properties. To the best of our knowledge, this is the first report of such NR–BC films.

## 2. Materials and Methods

The *Acetobacter xylinum* (AGR60) was isolated from nata de coco. The stock culture was kindly supplied by Pramote Tammarat, the Institute of Food Research and Product Development, Kasetsart University, Bangkok, Thailand. Natural rubber latex (NRL) with 60% dry rubber content (DRC) was purchased from the Rubber Research Institute of Thailand (RRIT, Bangkok, Thailand). Sucrose and ammonium sulfate were purchased from Ajax Finechem Pty Ltd (New South Wales, Australia). Acetic acid was purchased from Mallinckrodt Chemicals (Paris, KY, USA). Absolute ethanol was purchased from QRec (Chonburi, Thailand).

### 2.1. Film Preparation

For BC biosynthesis, the medium for the inoculum was coconut-water supplemented with 5.0% (w/v) sucrose, 0.5% (w/v) ammonium sulfate, and 1.0% (w/v) acetic acid. The medium was sterilized at 110 °C for 5 min. Precultures were prepared by a transfer of 50 mL stock culture to 1000 mL in 1500 mL bottle and incubated statically at 30 °C for 7 days. After the surface pellicle was removed, a 5% (v/v) preculture broth was added to sterile medium and statically incubated at 30 °C for five days in a Petri-dish. All sample BC pellicles were purified by washing with deionized water (DI) for 30 min and, then treated with 1% NaOH (w/v) at room temperature to remove bacterial cells for 24 h, followed by a rinse with water until the pH became 7.0. The BC pellicles were soaked in DI water and stored at 4 °C until use.

The procedure for the preparation of BC reinforced with NR (NR–BC) films was developed as follows. NRL was diluted with DI water to form NRL suspension with concentrations of 0.5%–10% dry rubber content (DRC) (expressed as weight per volume). In order to reduce the viscosity of NRL suspension, 6 mL of 50% (v/v) aqueous ethanol solution was slowly added into 300 mL NRL suspension. BC pellicle was then immersed in NRL suspension with concentrations of 0.5%–10% DRC for 48 h as the immersion temperature varied from 30–70 °C. Then, it was washed with DI water, air-dried at 30 °C for 48 h, and stored in plastic film at room temperature. BC was defined as the unmodified BC and NR–BC was defined as the modified BC by immersing in an NRL suspension. The xNR–BCy film was defined as the NR–BC film modified by immersing in an NRL suspension at x% DRC and at an immersion temperature of y °C. For example, 0.5NR–BC50 was the NR–BC film modified by immersing in an NRL suspension at 0.5% DRC and at a temperature of 50 °C.

### 2.2. Characterization 

#### 2.2.1. Field Emission Scanning Electron Microscopy (FESEM)

The examination of the surface morphology was performed by field emission scanning electron microscopy (FESEM). Scanning electron micrographs were taken with JEOL JSM-7610F microscope (Tokyo, Japan). The films were frozen in liquid nitrogen, immediately snapped, and vacuum-dried. Then, the films were sputtered with gold and photographed. The coated specimens were kept in dry place before the analysis. The FE-SEM was obtained at 10 kV, which was considered to be a suitable condition for these samples. The average thickness of the dried BC films and NR–BC films was measured using the ImageJ program.

#### 2.2.2. Laser Particle Size Distribution (PSD)

The particle sizes of rubber in NRL and NRL added with ethanol were investigated by laser diffraction technique. Particle size distribution curves were taken by Mastersizer 3000 (Malvern, UK). The operating size classes were recorded in the range of 0.01–3000 µm at a stirrer speed of 2000 rpm.

#### 2.2.3. Fourier Transform Infrared Spectroscopy (FTIR) 

The chemical structures of the films were analyzed and recorded by FTIR with a Nicolet SX-170 FTIR spectrometer (Thermo Fisher Scientific, Waltham, MA, USA) in the region of 4000–500 cm^−1^ at a resolution of 4 cm^−1^.

#### 2.2.4. Water Absorption Capacity (WAC) 

Water absorption capacity (WAC) was determined by immersing the pre-weight of dry films of 20 mm × 20 mm in DI water at room temperature (30 °C) until equilibrium. Then, the films were removed from water and blotted out with Kim wipes. The weights of the hydrate films were then measured, and the procedure was repeated until there was no further weight change. WAC was calculated by the following Equation (1):

(1) $$\mathrm{WAC}\%=\left[\frac{W_{h}-W_{d}}{W_{d}}\right]\times 100,$$

where *W_h_* and *W_d_* denote the weights of hydrate and dry films, respectively.

#### 2.2.5. Toluene Uptake (TU)

Specimens of BC and NR–BC films of 20 mm × 20 mm were weighed and immersed in toluene at room temperature. After that, the specimens were weighed. The procedure was repeated until there was no further weight change. The toluene uptake (TU) was calculated by the following Equation (2):

(2) $$\mathrm{TU}\%=\left[\frac{W_{t}-W_{0}}{W_{o}}\right]\times 100,$$

where *W_0_* and *W_t_* denote the weights of films before and after the immersion in toluene, respectively.

#### 2.2.6. X-Ray Diffraction (XRD) 

The examination of the crystal structures of the films was performed by X-ray diffractometer (model D8 Discover, Bruker AXS, Karlsruhe, Germany). The films were cut into strip-shaped specimens of 4 cm in width and 5 cm in length. The operating voltage and current were 40 kV and 40 mA, respectively. Samples were scanned from 5–40° 2θ using CuKa radiation.

#### 2.2.7. Differential Scanning Calorimetry (DSC)

DSC analysis was used to measure the thermal properties of the films, such as glass transition temperature (Tg) and crystalline melting temperature (Tm). A sample of about 4 mg was sealed in aluminum pan for DSC analysis under nitrogen gas. In addition, the curing behavior of the films was determined using a NETZSCH DSC 204 F1 Phoenix (Selb, Germany). The scanning range was −100 to 200 °C with a heating rate of 5 °C/min.

#### 2.2.8. Thermal Gravimetric Analysis (TGA)

The thermal weight changes of BC, NR, and the films were determined using TGA (Q50 V6.7 Build 203, Universal V4.5A TA Instruments, New Castle, DE, USA) in a nitrogen atmosphere. The scanning range was 30 °C to 700 °C with a heating rate of 10 °C/min. The initial weight of each sample was around 10 mg and percentage weight loss versus decomposition temperature by TGA analysis was determined.

#### 2.2.9. Mechanical Properties Testing

The tensile strength of the films was measured by Instron Testing Machine (ASD8-82A.TSX, NY, USA). The test conditions followed ASTM D882. The determination of elongation at break, tensile strength, and Young’s modulus was performed using films in strip-shaped specimens of 10 mm in width and 10 cm in length. The mechanical properties of each sample were the average values determined from five specimens. 

#### 2.2.10. Biodegradation in Soil

Biodegradation of BC and NR–BC films in soil for six weeks was evaluated. The samples were cut into square pieces of 3 cm × 3 cm and were buried in 10 cm soil depth under uncontrolled temperature (24–35 °C). Samples were taken out every week and washed with DI water. Then, the samples were dried at 50 °C for 24 h and weighed. The specific biodegradation rates based on the mass loss of films were determined by the following Equation (3):

(3) $$\mathrm{Biodegradation}\%=\left[\frac{W_{1}-W_{2}}{W_{1}}\right]\times 100,$$

where *W_1_* and *W_2_* denote the initial dry weight of the samples (g) and the residue dry weight of films after biodegradation in soil, respectively.

## 3. Results

### 3.1. NR Particle Size Distribution

**Figure 1:**
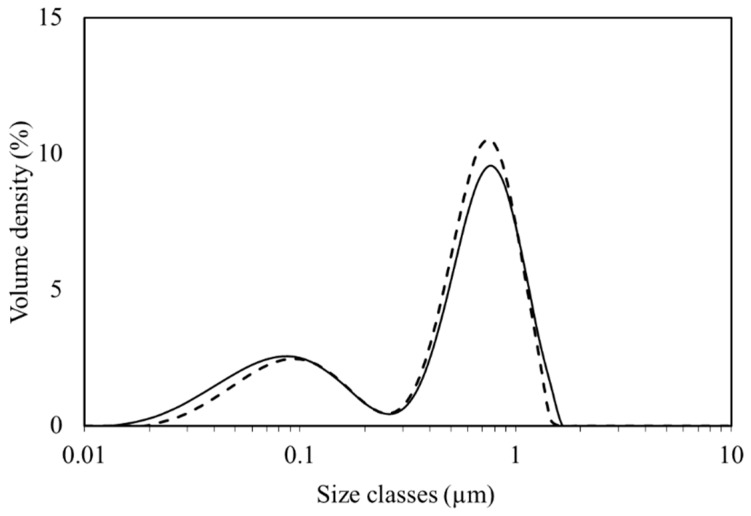
Particle size distribution of natural rubber (NR) in NR latex (NRL) suspension: in the absence of ethanol aqueous solution (solid line) and with the addition of ethanol solution (dash line).

The particle size distribution (PSD) of NR was analyzed by a laser diffraction particle size technique. The rubber particle size from NRL suspension varied from 0.01–2 µm, as shown in Figure 1. A bimodal PSD having two peaks at 0.08 µm and 0.7 µm was observed, with corresponding volume densities of about 2.6% and 9.6%, respectively. A solution of 50% (v/v) ethanol was slowly added into NRL suspension at 2.0% (v/v) to promote the penetration of NR into the BC nanofibrous network structure. No significant change in particle size distribution of NR in NRL suspension with the addition of the ethanol solution was observed.

### 3.2. Effects of NRL Concentration and Temperature on Integration of NR into BC

**Figure 2:**
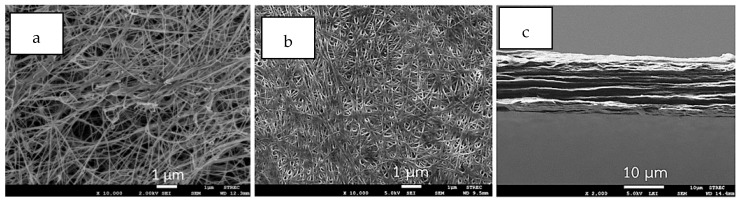
Field emission scanning electron microscopy (FESEM) images of surface morphologies of never dried films of bacterial cellulose (BC) (**a**) and dried BC films: surface (**b**) and cross section (**c**).

**Figure 3:**
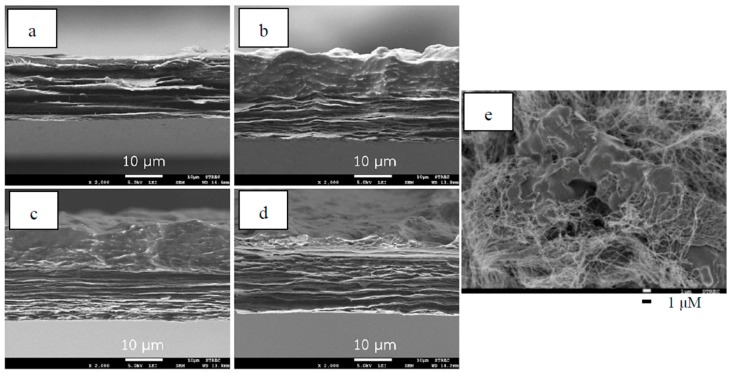
FESEM images of the cross section of dried films of 0.5NR–BC50 (**a**); 2.5NR–BC50 (**b**); 5NR–BC50 (**c**); 10NR–BC50 (**d**); and a closer look of the surface of dried NR–BC film (**e**).

**Figure 4:**
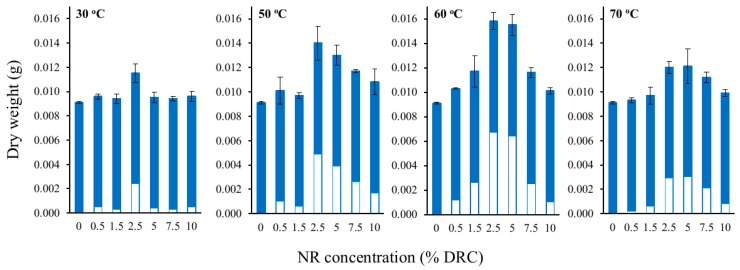
Dry weight of NR–BC films modified by immersing in an NRL suspension at various concentrations (0%–10% dry rubber content (DRC)) at immersion temperatures of 30 °C, 50 °C, 60 °C, and 70 °C. Values were expressed as mean ±SD (*n* = 3); blue bar = the dry weights of BC before the immersion and white bar = the estimated NR dry weights in the composites.

Surface morphology of a never-dried BC film is shown in Figure 2a. Surface and cross section of dried BC films are shown in Figure 2b,c, respectively. According to the observation from the FESEM images, the surface of never-dried BC films comprises microporous structures of fibrous networks with nanocellulose fiber diameters of ~50–100 nm. Never-dried BC films possess pore sizes ranging between 0.1–2 µm located between the fibers. A similar pore structure of unmodified BC hydrogel was previously reported [18]. After air drying at 30 °C, a dense nanocellulose layer structure was obtained, as shown in Figure 2b. Water loss during drying could result in shrinkage and a compact structure. The cross section of dried BC films in Figure 2c shows that the structures are composed of multilayers of thin sheets, which is the characteristic feature of BC film previously reported [13]. During the immersion, NR diffused into and gradually filled the never-dried BC pores, and by doing so, might coat the surface. FESEM images of the cross section of the dried NR–BC films by immersion in NRL suspensions of 0.5%, 2.5%, 5%, and 10% DRC at 50 °C are shown in Figure 3. The NR–BC films comprise dense nanocellulose layers incorporated with NR. The amount of integration of NR into BC was in association with the increase in weight (Figure 4) and thickness of the dried films (Table 1). The accumulated NR in the composite was estimated from the change of dried weight of the films before and after the imersion in NRL suspension, as shown in Figure 4. The average weight of the dried BC film was 0.0091 ± 0.0001 g, whereas the diameter and thickness were 14.01 ± 0.13 cm and 12.05 ± 0.26 µm, respectively. The diffusion and integration of NR into BC increased as a function of NRL concentration up to around 2.5%–5% DRC. The high amount of NR accumulated in the composite films was obtained by the immersion in NRL suspension at 2.5% DRC (at 50 °C), 5% DRC (at 50 °C), 2.5% DRC (at 60 °C), and 5% DRC (at 60 °C), where the estimated ratios of NR/BC in the composite films were 53.9%, 42.9%, 73.6%, and 70.3%, respectively (the amounts of NR in the composite films were 35.0%, 30.0%, 42.4%, and 41.3%, respectively). However, further increasing the NR concentration in the suspension to 7.5% and 10% DRC resulted in a significant decrease of NR adsorption. Higher weights and thicknesses of films were also observed as a function of increased immersion temperature from 30 °C to 50–60 °C. The rate of diffusion of NR into nanocellulose fiber networks should generally increase with temperature up to a certain point as a result of increased kinetic energy. Similar changes to film thickness and surface area have previously been reported in relation to the modification of BC by impregnation with aqueous alginate solutions at 1%–3% (w/v) at the immersion temperature of 30 °C to 50 °C [19].

### 3.3. Mechanical Properties

**Figure 5:**
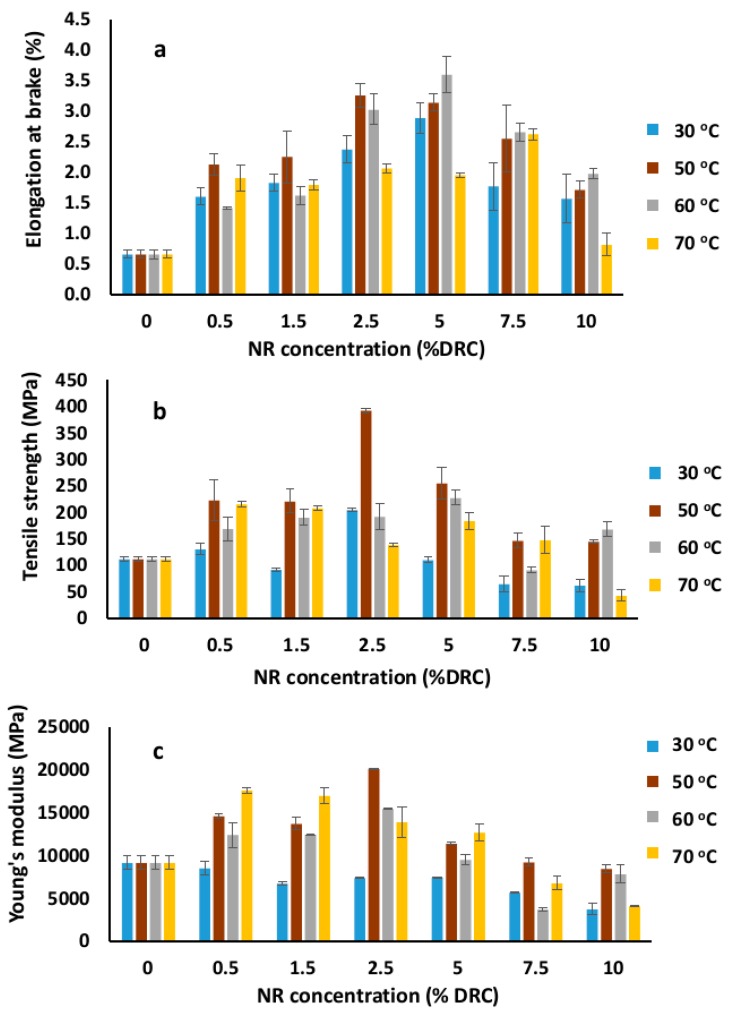
Elongation at break (**a**), tensile strength (**b**), and Young’s modulus (**c**) of the dried BC and NR–BC films modified by the immersion in NRL of various concentrations (0%–10% DRC) at various immersion temperatures.

The mechanical properties of BC and NR–BC films were analyzed in terms of elongation at break, tensile strength, and Young’s modulus, as shown in Figure 5. According to our previous studies, the tensile strength, elongation at break, and Young’s modulus of unmodified BC films could be varied around 70–300 MPa, 0.5%–5%, and 5–17 GPa, respectively, depending on many factors, such as culture conditions, drying conditions, and bacteria strain [19,20,21]. In this study, the unmodified BC film possessed an elongation at break, tensile strength, and Young’s modulus of 0.6%, 112.4 MPa, and 9.14 GPa, respectively; meanwhile, the corresponding values for the uncured NR film developed from pure NRL were around 100%–111%, 0.8–1.2 MPa, and 1.6–2.4 MPa, respectively [17,22]. The NR–BC films have considerably higher elongation at break compared with the unmodified BC films. The maximum values of elongation at break, at 3.0%–3.5%, were obtained when immersing BC in NRL at a concentration range of 2.5%–5% DRC between 50 and 60 °C. The incorporation of NR into the BC matrices resulted in the ability of the fibers to absorb more energy, and thus prevent the nanocellulose fibers from breaking. Consequently, when compared with the BC films, the films were demonstrated to elongate more prior to breaking. The NR–BC film tensile strengths are also significantly higher when compared with those of the BC-only film (Figure 5b. The optimal conditions to achieve maximum tensile strength of the NR-BC films are during the preparation of 2.5NR–BC50, in which the tensile strength increased to 392.4 MPa (a ~3.5-fold increase over that of the unmodified BC film). Additionally, the films treated with NRL suspension of 5% DRC and at the immersion temperature of 50–60 °C exhibited considerably higher tensile strengths when compared with the unmodified BC film. The tensile strengths of 2.5NR–BC50, 2.5NR–BC60, 5NR–BC50, and 5NR–BC60 showed a 3.5-, 1.7-, 2.3-, and 2.0-fold increase, respectively, over that of the unmodified BC film. The Young’s modulus of pure BC and the films are shown in Figure 5c. The unmodified BC film exhibits a Young’s modulus of 9.14 GPa, whereas the NR–BC films display higher Young’s modulus values. Under the optimal preparation conditions, 2.5NR–BC50 displayed a Young’s modulus value of 20.05 GPa or an ~2-fold increase over that of the BC film. As the maximum enhancement of the mechanical properties was obtained by immersing at 50 °C, the results of the following studies were performed on NR–BC films prepared by immersing in NRL suspensions at 50 °C.

### 3.4. FTIR Analysis

**Figure 6:**
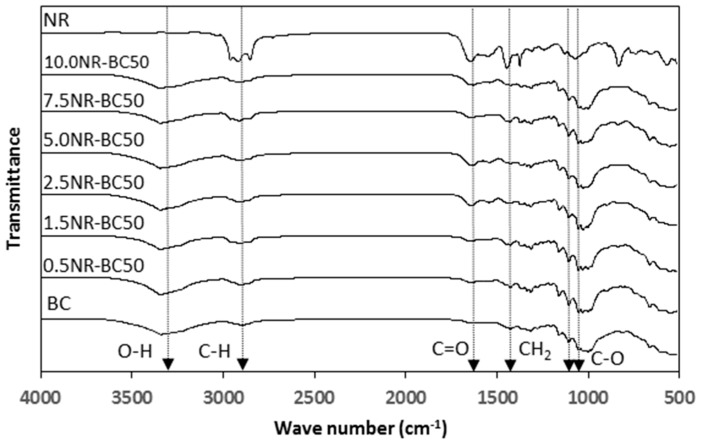
Fourier transform infrared spectroscopy (FTIR) spectra of BC, NR, and NR–BC films treated by the immersion in NRL of various concentrations at 50 °C.

As shown in Figure 6, the FTIR spectrum of pure BC shows characteristic peaks at 3347 cm^−1^ attributed to O–H stretching vibrations from hydroxyl groups, and at 2900–2800 cm^−1^ corresponding to C–H stretching, while a peak at 1650–1640 cm^−1^ corresponds to the vibration of the carbonyl group (C=O). A peak at 1440 cm^−1^ is attributed to CH_2_ bending, and a peak located at 1165–1060 cm^−1^ is attributed to C–O stretching [17,23,24]. The FTIR spectrum of NR reveals several characteristic peaks. The peak at 2960 cm^−1^ is attributed to the vibration of C–H stretching, while the peak located at 2917 cm^−1^ corresponds to the symmetric stretching of methylene (−CH_2_). The asymmetric stretching of the CH_3_ group is observed at 2847 cm^−1^. A peak located at 1637 cm^−1^ is assigned to the vibration of C=C, while the peak located at 1465 cm^−1^ is attributed to the symmetric bending of CH_2_. The peak at 1375–1450 cm^−1^ is attributed to the vibration of CH_2_ asymmetric bending and stretching [17,25]. The FT-IR spectra of the NR–BC films display characteristic peaks of both BC and NR. No occurrence of new peaks was observed.

### 3.5. X-Ray Diffraction (XRD) Analysis

**Figure 7:**
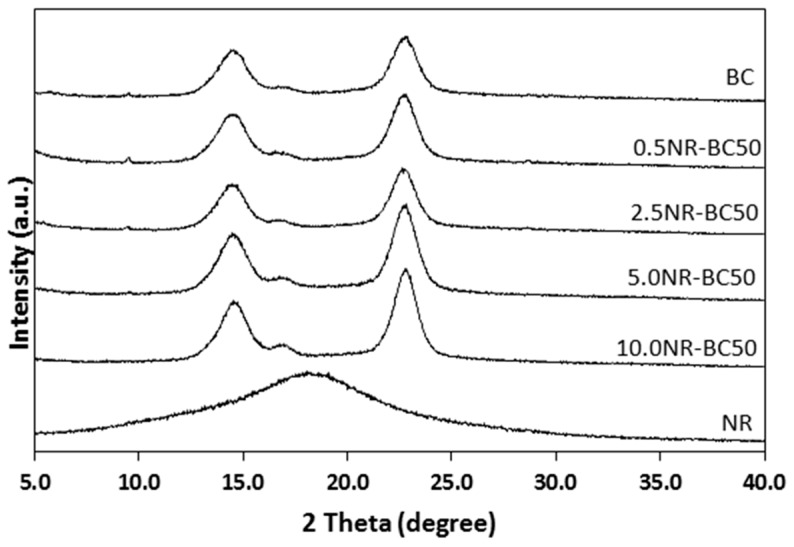
X-ray diffraction (XRD) patterns of BC, NR, and NR–BC films treated by the immersion in NRL of various concentrations at 50 °C.

The XRD patterns of BC and the NR–BC films are shown in Figure 7. The characteristic peaks of BC comprise three main peaks at two theta angles of 14.5°, 17.0°, and 22.8°, associated with the typical profile of cellulose [26]. The diffraction patterns of the NR–BC films exhibit a higher degree of order, as seen by the sharp peaks, whereas the diffraction pattern relating to the BC-only film displays relatively broad peaks, indicating a less crystalline material. The NR film displays a typical diffraction pattern of an amorphous polymer having a prominent broad hump located at a two-theta angle of 18°. The NR–BC films, prepared at various concentrations and immersion temperatures, display the same characteristics of the BC-only film diffraction pattern, indicating the presence of the BC crystalline structure. The peaks at two-theta ≈ 17° and 22.8° became slightly more intense with increasing NR, which might imply that the integration of NR into fibrous structure of BC might have some effect on the crystalline structure of BC. The amorphous broad hump associated with NR was not observed in the XRD patterns, as there was a small proportion of NR in the BC matrices.

### 3.6. Differential Scanning Calorimetry (DSC)

**Figure 8:**
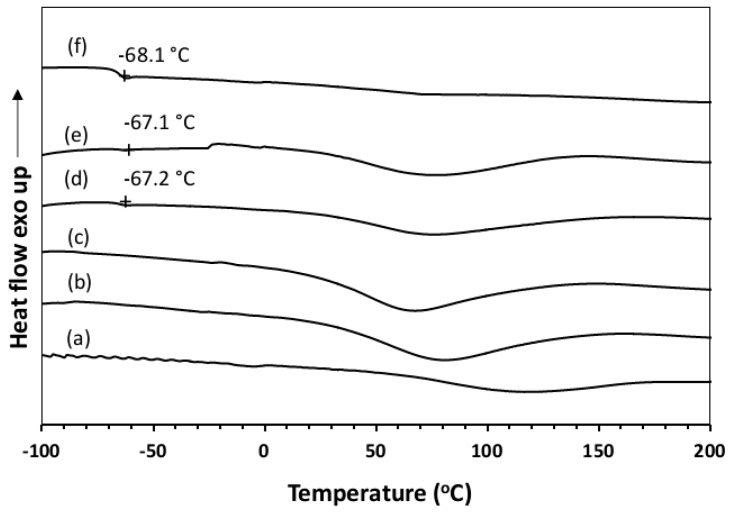
Differential scanning calorimetry (DSC) chromatograms of BC (**a**), NR (**f**), and NR–BC films of 0.5 NR–BC50 (**b**), 2.5 NR–BC50 (**c**), 5.0 NR–BC50 (**d**), and 10.0 NR–BC50 (**e**).

The DSC thermograms of dried BC, NR, and the NR–BC films are shown in Figure 8. The glass transition temperature (Tg) of pure BC was barely detected because the highly crystalline structure of nanocellulose exhibited flat heat flow curves, which are difficult to separate from the baseline. For the thermal degration, the BC film displays two exothermic broad peaks. The first peak around 217 °C is associated with the thermal degradation of proteinaceous matter in the BC film [19,26]. The second peak at ~304 and 340 °C is ascribed to the partial pyrolysis of cellulose [27]. The DSC thermogram of NR exhibited a Tg at −68.1 °C. The DSC thermograms of 5.0NR–BC50 and 10.0NR–BC50 exhibited a Tg at around −67 °C, slightly higher than the Tg of NR. The endothermic peaks exhibited by the NR–BC films between 30–150 °C are attributed to water loss or dehydration of the films [19,28]. NR loading into the BC matrices (2.5NR–BC50, 5.0NR–BC50, and 10.0NR–BC50) resulted in a slight change to the position of the exothermic peaks.

### 3.7. Thermal Gravimetric Analysis (TGA) and Differential Thermal Analysis (DTA)

**Figure 9:**
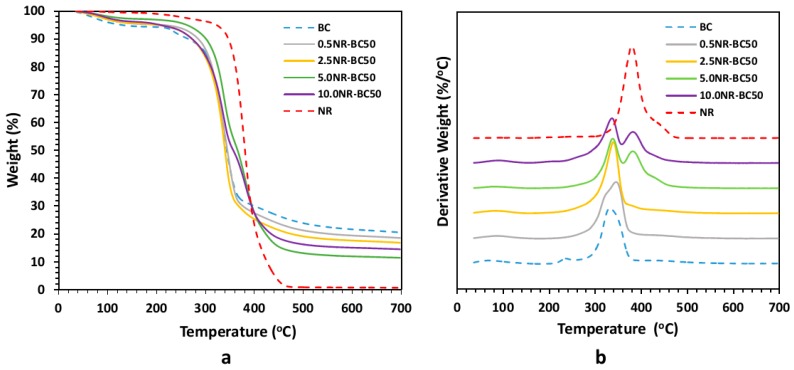
Thermal gravimetric analysis (TGA) (**a**) and differential thermal analysis (DTA) (**b**) curves of BC, NR, and NR–BC films.

The TGA and DTA curves of BC, NR, and the NR–BC films are shown in Figure 9a,b, respectively. The slight weight loss from 30–200 °C is associated with the vaporization of water. The percentage weight loss of pure BC across a temperature range of 210–240 °C is ~6 wt.%, indicating the decomposition of proteins [29]. The major pyrolysis of BC, resulting from the decomposition of cellulose [30], occurs at ~300–360 °C, and results in a residual char product (≈20 wt.%). Conversely, the pyrolysis temperature of NR occurs at ~340–440 °C, at which about half of the mass loss is observed for pyrolysis at 380 °C. The thermal decomposition of the NR–BC films is divided into three weight loss stages. At temperatures <200 °C, the weight loss is associated with water loss. The second and third weight loss stages occur across a temperature range of 300–400 °C. The maximum rate of weight loss of the films occur at ~320 °C and ~380 °C in accordance with the decomposition of BC and NR, respectively. Films of 5.0NR–BC50 and 10.0NR–BC50 presented slightly increased thermal stability when compared with the BC film and other NR–BC films.

### 3.8. Water Absorption Capacity (WAC)

**Figure 10:**
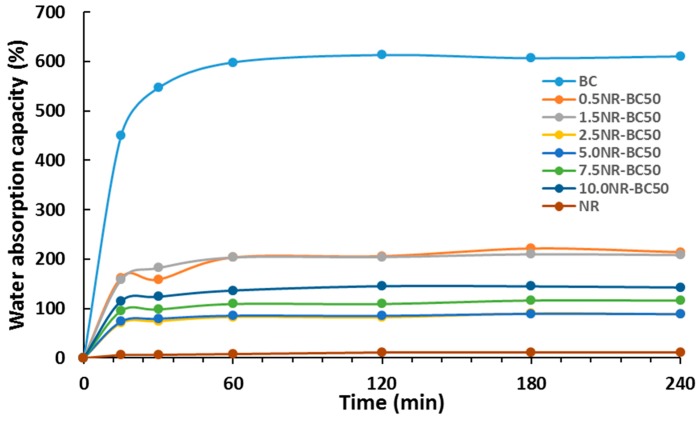
Water absorption capacity (WAC %) with time of NR–BC50 films.

Water absorption capacities of BC, NR, and NR–BC films treated by the immersion in NRL of various concentrations at 50 °C are shown in Table 2 and Figure 10. Overall, all films showed rapid adsorption of water in the first 20 min and then slow adsorption until concentrations reached equilibrium at around 1 h. The BC film has highly water absorption capacity at 610.5% because of the hydrophilic property of the hydroxyl group in its structure [31,32]. Because of the hydrophobic structure of NR, the water absorption capacity of the NR film was very low (10.9%). As compared with BC, NR–BC films had significantly higher resistance to water.

### 3.9. Toluene Uptake (TU)

NR is a nonpolar material; therefore, it is soluble in non-polar solvents. Toluene is an aromatic solvent that is mostly used in many rubber industries. Thus, the effect of toluene uptake on NR-BC films was investigated (Table 2). At initial absorption, the toluene uptake of the NR film rapidly increased and reached the maximum value at 2642% in 1 h. After that, the degradation of the NR film by dissolving in toluene was observed [33]. Because of its hydrophilic nature, the BC film had high resistance to non-polar solvents and exhibited very low toluene uptake at around 6.4%. The toluene uptake of NR–BC films is higher than BC film, but so much less than NR (0.02–0.05 of NR film).

### 3.10. Biodegradation in Soil

**Figure 11:**
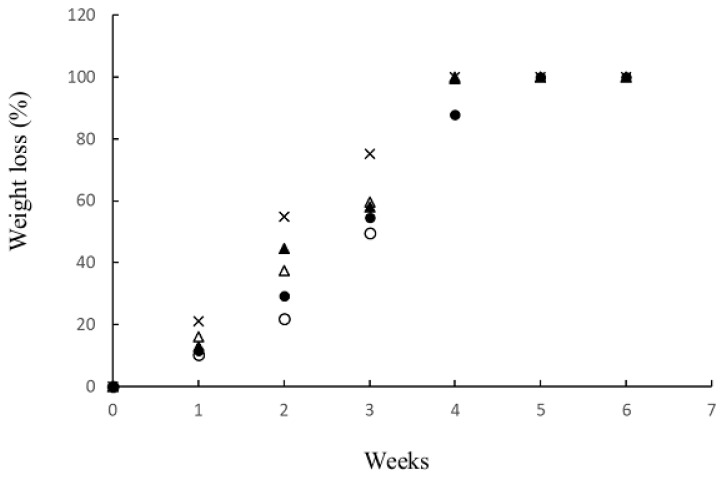
Biodegradability of films in soil after 0–6 weeks. (BC = ×; 0.5 NR–BC50 = white triangle; 2.5 NR–BC50 = white circle; 5 NR–BC50 = black circle; 10 NR–BC50 = black triangle).

**Figure 12:**
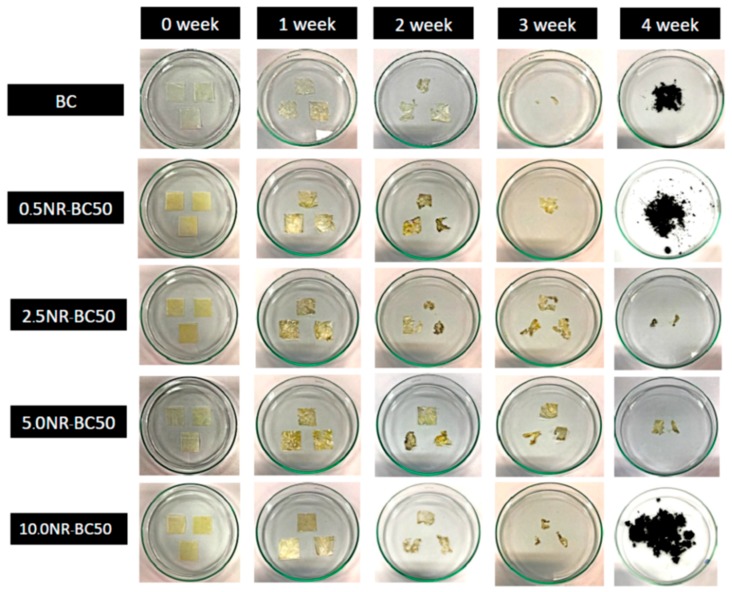
The images of degraded materials at the different weeks in the study of biodegradation in soil.

Biodegradability is an essential property when considering environment issues, and is a critical property for the application of green packaging materials. BC structures comprise crystalline nanocellulose fibers and a minor amount of amorphous cellulose chains, which can be attacked by multiple microorganisms in the soil through enzymatic degradation [34,35]. The conformation of NR is relatively resistant to biodegradation through microorganisms when compared with many other natural polymers [36]. However, there are known microorganisms in soil such as bacteria and fungi that have the ability to degrade NR [36,37]. Natural latex rubber is biodegradable, as is claimed by numerous products and manufacturers. Natural rubber latex gloves can be disposed of by either landfill or incineration, which are not harmful to the environment. Recently, it was shown that the mixed culture isolated from soil samples collected from rubber contaminated ground in Songkhla province, Thailand had potential in degrading rubber, in which significant changes could be detected within 30 days [38]. In this study, the biodegradability of BC and the NR–BC films in soil is shown in Figure 11 and Figure 12. The films underwent soil burial experiments and the average soil temperature was 35.1 ± 2.0 °C. The BC film exhibited a higher weight loss percentage when compared with the other films, and was completely decomposed within four weeks. The NR–BC films were completely decomposed within 4–6 weeks. Overall, higher NR loadings were observed to slow the decomposition rate of the films. Films of 2.5NR–BC50 and 5NR–BC50 demonstrated a higher resistance to microorganism degradation when compared with 0.5NR–BC50 and 10NR–BC50. The films of 2.5NR–BC50 and 5NR–BC50 were completely decomposed within six and five weeks, respectively, whereas 0.5NR–BC50 and 10NR–BC50 were completely decomposed within four weeks.

## 4. Discussion

BC pellicle was immersed in diluted NR latex (NRL) suspensions with the supplement of ethanol aqueous solution. The slow addition of a 50% (v/v) ethanol solution into the NRL suspension at 2.0% (v/v) did not show a significant effect on NR particle size. However, it could result in a decrease in viscosity of the NRL suspension as a result of the reduction of the gel content of rubbers from NRL [39]. It was found that the addition of ethanol in NRL at a specific fraction could promote the penetration of NR into the BC nanofibrous network structure. However, as ethanol is an organic polar solvent, the addition at too high a fraction into the NRL suspension resulted in the coagulation of NR molecules [40]. From our preliminary test, the addition of ethanol at concentration ≥70% v/v into NRL for 2.0% (v/v) or the addition of 50% v/v ethanol into NRL for ≥3.0% v/v caused coagulation of NR.

After the modification by immersing BC films in diluted NRL suspensions, it was found that the maximum dry weight of the NR–BC films was around 0.014–0.016 g, which was about 1.5–1.8 of that of the BC film. The maximum thickness of the NR–BC films was 20–27 µm, or about 1.7–2.3 of that of the BC film. The maximum amount of integration of NR into BC was obtained by immersing BC in NRL suspensions of 2.5%–5.0% DRC at an immersion temperature of 50–60 °C. However, further increasing the NRL concentration above 5.0% DRC led to the agglomeration of NR molecules, resulting in a lower diffusion of NR molecules into the BC film. At higher concentrations, the close vicinity of the NR molecules with respect to each other could result in increased interaction and agglomeration. As the BC pellicle pore size was relatively small, the diffusion of agglomerated NR into the BC fibrous network was hindered. Considering how diffusion is influenced by immersion temperature, at low NRL concentrations (< 2.5% DRC), no significant increase of NR–BC film thickness was observed when increasing the immersion temperature from 30 °C to 70 °C. At low NR concentrations, low adhesion of NR to the BC matrices was observed. When BC was immersed in NRL suspensions at medium to high concentrations (2.5%–10% DRC), the NR–BC film thickness significantly increased as a function of increased immersion temperature from 30 °C to 60 °C. Increased kinetic energy of the NR molecules at high temperature was considered to promote the diffusion of NR into the BC matrices. At high temperature, the agitated particles were subjected to stronger and more frequent collisions. However, NR–BC film thickness and weight decreased as a function of elevated immersion temperature from 60 °C to 70 °C, which implied less accumulation of NR in BC films. When subjected to a high immersion temperature of 70 °C, the NR molecules, as a result of a high collision rate, could form particle agglomeration. The coating of agglomerated NR on the BC surface and in the BC fibrous networks was thought to prevent the diffusion of small NR particles into the BC pores. The low degree of NR penetration resulted in a smaller NR–BC film thickness when the immersion temperature increased from 60 °C to 70 °C.

BC films usually possess a high mechanical strength (high modulus and tensile strength), but low elongation at break (or low fracture strain). Conversely, NR films show higher elongation at break, but possess a relatively lower mechanical strength as compared with BC films. In this study, it was shown that the mechanical properties of the BC films were considerably improved by the addition of NR into BC matrices. The important factors that affect the diffusion of NR into BC matrices are the concentration of NRL suspension and temperature. The concentration of NRL suspension at 2.5%–5.0% DRC and the immersion temperature at 50–60 °C are the optimal conditions for high diffusion of NR into BC matrices. The integration of NR into BC matrices resulted in improved mechanical properties of the films. When subjected to a high immersion temperature of 70 °C, the NR molecules, as a result of a high collision rate, could form particle agglomeration. The agglomerated NR particle could cover some parts of the BC surface and filled in the pores of BC matrices. The large particles could prevent the diffusion of small NR particles into the pores of the inner part. At high temperature, NR agglomeration could be generated to a greater extent, especially in the condition with higher concentration of NRL suspension. This agglomeration might cause a problem of poor distribution of NR inside the BC matrices. As a result, at an immersion temperature of 70 °C, NR–BC films exhibited the largest Young’s modulus in the 0.5% DRC group, but exhibited the smallest Young’s modulus in the 10% DRC group. However, under the optimal condition, NR integrated into the BC films and was well distributed in BC matrices. NR could bind the nanocellulose fibers together and, consequently, the mechanical properties of the NR–BC films, with respect to their modulus and strength, were enhanced compared with the BC film. NR possessed high structural regularity and typically crystallizes spontaneously when stretched [41]. The NR bonds in the nanocellulose fibrous network restricted the movement of the nanocellulose fibers and enhanced mechanical strength [42]. It was revealed that the presence of NR on the BC matrices in the NR–BC films induced superior mechanical properties such as high elongation at break and high tensile strength. Therefore, the combination of a nanocellulose fibrous network and NR could result in a synergistic effect on the mechanical properties. 

The FTIR and XRD results showed that there was no chemical interaction between NR and BC; however, the integration of NR into fibrous structure of BC might improve crystalline structure of BC. At high NR diffusion into BC fibers, NR–BC films exhibit relatively high structural and thermal stability. It was shown that 5.0NR–BC50 and 10.0NR–BC50 presented relatively increased thermal stability when compared with that of the BC film and the other NR–BC films. On the other hand, it was shown that XRD peaks of 5.0NR–BC50 and 10.0NR–BC50 are also slightly sharper than the others. Therefore, the integration of NR into BC matrices at a certain content (an optimal concentration range) might have some positive effects on the crystalline structure of BC. The crystalline structure could affect thermal properties of the composites. The result of TGA residual mass (Figure 9) showed that the remaining mass by the higher order was BC > 0.5 NR–BC50 > 2.5 NR–BC 50 > 10NR–BC50 > 5.0 NR–BC 50 > NR. According to our previous study [17], the char yield of BC was higher than that of NR, and the char yields increased along with the ratio of BC in NR composites. In this study, the ratio of BC/NR by the higher order was 0.5NR–BC50 > 10 NR–BC50 > 5.0NR–BC50 > 2.5NR–BC50 (Figure 4). Compared with the other composite films, the remaining mass of the composite film of 0.5 NR–BC50 was the highest because the ratio of BC/NR of this composite film was higher than the others. For the same reason, the residual of 10.0NR–BC50 was greater than that of 5.0NR–BC50. However, it is noticed that the remaining mass of 2.5 NR–BC50, which has the highest ratio of NR (or the lowest ratio of BC) is quite high when compared with the others. As a result, it was suggested that not only the composition, but also the structure of the composites could also have an effect on the thermal properties. 

BC has a hydrophilic structure and NR has a hydrophobic structure; therefore, the values of WAC% decreased with the ratio of NR in the NR–BC composites. The films of 2.5NR–BC50 and 5NR–BC50 had the highest water resistance (the lowest WAC%). Because of NR binding into BC fibers, only small amounts of water could diffuse or be adsorbed into NR–BC films. Consequently, the water resistance of NR–BC films increased with NR concentration in the films. The toluene uptake (TU%) of NR–BC50 films was in range of 30%–70% and the increase in toluene uptake was related to the concentration of NR in the films. However, the toluene uptake of NR–BC films was much lower than that of NR films and, after the immersion of NR–BC films in toluene for 4 h, no significant change in overall outlook of the NR–BC films was observed. The good resistance to toluene should be attributed to the hydrophilic nature and high stability of nanocellulose network structure of BC in nonpolar solvents. 

The evaluation of biodegradation capability of NR–BC50 films in comparison with BC films was conducted in soil environment for six weeks. BC fiber is biodegradable by various bacteria and fungi in soil. The microorganism first attacks the nanocellulose amorphous region and, thereafter, decomposes all the crystalline regions. On the other hand, it has been previously reported for a slow process of biodegradation of NR and related compounds by some microorganisms in soil [43]. In this study, during the biodegradation test, the NR–BC films transitioned to a loose structure. The films were lumpy and highly stretched as a result of the decomposition of nanocellulose by the microorganisms, while the NR particles in the NR–BC films underwent a relatively slower rate of decomposition than the nanocellulose of BC. However, all NR–BC films were biodegradable and could be degraded completely in soil environment within 5–6 weeks.

## 5. Conclusions

Films of BC reinforced with NR, prepared by immersing BC into a diluted NRL suspension, demonstrated superior mechanical properties when compared with BC-only films. The combination of a nanocellulose fibrous network and NR polymer synergistically improves the film mechanical properties. Films of 2.5NR-BC50 demonstrated considerably enhanced tensile strength and elongation at break. The NR–BC films also exhibit high structural and thermal stability and are completely degraded in soil within 5–6 weeks.

## Figures and Tables

**Table 1:** Thickness of natural rubber (NR)–bacterial cellulose (BC) films.

Samples	Thickness (µm)	Samples	Thickness (µm)
0.5NR–BC30	12.74 ± 0.58	0.5NR–BC60	13.86 ± 0.13
2.5NR–BC30	19.08 ± 0.38	2.5NR–BC60	24.97 ± 0.90
5NR–BC30	15.85 ± 0.53	5NR–BC60	27.21 ± 0.39
10NR–BC30	14.66 ± 0.49	10NR–BC60	24.15 ± 1.62
0.5NR–BC50	14.84 ± 0.64	0.5NR–BC70	13.32 ± 0.58
2.5NR–BC50	20.61 ± 0.24	2.5NR–BC70	20.98 ± 0.69
5NR–BC50	21.99 ± 0.96	5NR–BC70	25.27 ± 0.29
10NR–BC50	18.54 ± 0.63	10NR–BC70	21.73 ± 0.35

Values are expressed as mean ±SD (*n* = 3).

**Table 2:** Water absorption capacity (WAC%) and toluene uptake (TU%) of NR–BC50 films.

Solvent	Absorption (WAC%, TU%)							
	BC	0.5 NR–BC	1.5 NR–BC	2.5 NR–BC	5 NR–BC	7.5 NR–BC	10 NR–BC	NR
Water	610.5 ± 2.6	213.9 ± 6.4	208.2 ± 2.4	88.4 ± 3.3	88.6 ± 1.8	115.9 ± 3.3	142.5 ± 1.2	10.9 ± 0.3
Toluene	8.7 ± 1.0	33.2 ± 6.8	34.2 ± 12.6	70.9 ± 24.0	68.6 ± 13.5	30.5 ± 11.7	47.3 ± 3.4	2,652.8 ± 300.0

Values are expressed as mean ±SD (n = 3).
